# A Large Specific Deterrent Effect of Arrest for Patronizing a Prostitute

**DOI:** 10.1371/journal.pone.0000060

**Published:** 2006-12-20

**Authors:** Devon D. Brewer, John J. Potterat, Stephen Q. Muth, John M. Roberts

**Affiliations:** 1 Interdisciplinary Scientific Research, Seattle Washington, United States of America; 2 Independent Consultant, Colorado Springs Colorado, United States of America; 3 Quintusential Solutions, Colorado Springs United States of America; 4 University of New Mexico Albuquerque, New Mexico, United States of America; Université de Toulouse, France

## Abstract

**Background:**

Prior research suggests that arrest, compared with no police detection, of some types of offenders does not decrease the chances they will reoffend.

**Methodology/Principal Findings:**

We assessed the specific deterrent effect of arrest for patronizing a street prostitute in Colorado Springs by comparing the incidence of arrest for clients of prostitutes first detected through public health surveillance with the incidence of rearrest for clients first detected by police arrest. Although these sets of clients were demographically and behaviorally similar, arrest reduced the likelihood of a subsequent arrest by approximately 70%. In other areas of the United States, arrest did not appear to displace a client's patronizing.

**Conclusions/Significance:**

Our results suggest that apprehending clients decreases their patronizing behavior substantially.

## Introduction

Criminologists have long studied the effect that penalties for criminal behavior have on the subsequent offending of those penalized. This focus on specific deterrence has included evaluations of the impact of incarceration [1]–[4], fines [2], restitution [5], and other penalties [6], [7] among charged or convicted offenders. Researchers have also investigated the specific deterrent effect of arrest, compared with less severe interventions, such as warnings, on offenders who have been contacted by the police about their apparent criminal behavior [8]–[10].

Yet surprisingly little is known about the specific deterrent effect of arrest relative to no contact by the police. It is widely assumed that, even for minor crimes, offenders who are arrested are less likely to reoffend than those who escape police detection. Past research, though, indicates that arrest of juvenile and young adult offenders, compared with no police detection, may have no specific deterrent effect, and may even have a slight escalatory effect, on subsequent delinquent and criminal behavior generally [11]–[14] and for marijuana-related offenses in particular [15]. However, only young offenders and a limited range of offenses have been examined in prior work on this dimension of specific deterrence. In this investigation, we aim to improve understanding of this fundamental aspect of crime control by assessing the specific deterrent effect of arrest—relative to no police detection—for patronizing a prostitute.

## Methods

We compared the annual incidence rates of arrest for patronizing a prostitute for two groups of clients of prostitute women identified in Colorado Springs, Colorado, between 1970 and 2000. One group included clients first detected through arrest by the police; the other included clients first detected through public health activities and research. If the two groups were otherwise comparable, a lower arrest rate for clients first identified through arrest would imply that arrest has a specific deterrent effect on patronizing. We obtained the data on these two groups when the second and third authors directed the sexually transmitted disease control program of the local health department. The Colorado Springs police provided data on clients arrested for patronizing. Arrests constitute direct evidence of patronizing. Therefore, by using arrest data, we avoided the well-established problem of clients underreporting their patronizing activity in surveys [16]–[22].

### Police surveillance of clients

Clients identified by the police were all men arrested for patronizing, typically caught in stings in which female police officers posed as decoys. In Colorado Springs, as elsewhere in the US, client stings were conducted on the street in areas of high prostitution activity, as determined by complaints from community members and locations of prostitute arrests (Brewer et al., unpublished data). There is little a client can do to detect a decoy or avoid arrest once a negotiation for a sex act and price has been completed; similarly, police exercise very little discretion or control over which clients are ultimately arrested. Consequently, arrested clients approximate a representative sample of clients of street prostitute women, weighted by frequency of patronizing activity. Colorado Springs police indicated that virtually all arrested clients were convicted, and that sentences typically involved fines and/or probation. Therefore, arrest involved little or no physical incapacitation beyond the arrest episode.

For police-detected clients, the year of detection was the year of arrest. (Year of arrest was unknown for 398 clients who were arrested at some point before 1995, but none were rearrested, so their exclusion inflates our estimate of the rearrest rate for police-detected clients). One factor may depress our estimates of police-detected clients' rate of rearrest very slightly. A decoy's usual term of service in client stings was approximately 2 years and 5–8 decoys served at any one time. Therefore, if an arrested client continued patronizing unabated after arrest, recognized a decoy working a subsequent sting, and consequently avoided her, he would be, on average, 13–20% less likely to be rearrested during the first year after his initial arrest than if he had not recognized the decoy. However, the impact of this potential circumstance on estimation of police-detected clients' rearrest rate would be quite small given the long period of observation following each client's first arrest and decoys' comparatively short terms of service. Furthermore, Colorado Springs police reported to us that some arrested clients had prior contact with decoys in non-vice situations and yet still solicited the same officers as decoys. We also have found instances of clients being arrested multiple times by the same decoy in patronizing arrest data from other communities in the US (see section on “Data sets for assessing displacement” for a description of some of these data sets).

### Public health surveillance of clients

Public health surveillance of clients occurred between 1985 and 2000 and focused on clinic-based HIV testing and a study of local prostitutes, drug injectors, and their close personal contacts, including sex partners. Clients identified through HIV testing were men who acknowledged having sex with a prostitute since 1978. Ninety-six percent of clients detected through HIV testing had either voluntarily sought testing or were screened at the recommendation of a health care provider. The others were tested as part of HIV contact tracing efforts (locating, counseling, and testing sex and needle-sharing partners of HIV-infected persons) or in response to court orders (none connected to patronizing arrests). We excluded those clients identified from HIV testing who reported ever having just male sex partners or whose records indicated they had sex with male prostitutes. The locale in which clients patronized was recorded only in the first few years of HIV testing. We excluded clients who reported patronizing only outside of Colorado Springs. We included all other clients identified through HIV testing in many analyses, even though some may have patronized only outside of the local area.

In the study of prostitutes, drug injectors, and their contacts, local clients were recruited between 1988 and 1992 from the county STD and HIV clinics, outreach in areas of prostitution, and jail, and also were identified by other respondents [23]. Self-reported clients were men who acknowledged having sex with a local prostitute woman in the last 5 years (nearly all of whom reported patronizing within the 6 months before the first time they were interviewed).

For public health-detected clients, year of first detection was year of last reported patronizing (for HIV testing patients whose records included this information), year of interview or third-party identification (for study participants or those identified by them), or year of HIV testing (for HIV testing patients whose records did not specify date of last patronizing). Two clients who tested for HIV reported last patronizing in the same year they were arrested. These clients were conservatively coded as being first detected by the police. Nine other clients who tested for HIV reported last patronizing 5 or more years before their tests. We excluded these clients from our analyses because they appeared to be former clients only.

### Data sets for assessing displacement

Displacement of patronizing behavior from one jurisdiction to another and from the street to the off-street sector of prostitution subsequent to arrest would lead to a lower arrest rate for police-detected clients than public health-detected clients, with all other factors held constant. Hence, displacement must be assessed to interpret any difference in arrests rates between the two sets of clients. There were no data available to examine displacement for clients arrested in Colorado Springs. However, to measure geographic displacement, we obtained statewide prostitution arrest records for Texas (from the Department of Public Safety), Virginia (from the Department of State Police), Connecticut (from the Connecticut State Police), and Washington state (from various local jurisdictions) that indicated the jurisdiction of arrest and jurisdiction of arrestee residence. Because Texas does not have a patronizing-specific prostitution charge, we defined clients as males arrested for prostitution on dates in which 5 or more males were arrested within the same jurisdiction (presumably reflecting clients arrested in stings). We assessed the validity of this rule in arrest data from 8 large jurisdictions elsewhere in the US (Albuquerque, NM; Bronx County, NY; Indianapolis, IN; Kings County [Brooklyn], NY; Minneapolis, MN; New York County [Manhattan], NY; Queens County, NY; Seattle, WA) which indicated whether an arrestee bought or sold sex. Excluding Manhattan, we found that between 91–97% (median = 94%) of males arrested in these jurisdictions on dates in which 5 or more males were arrested on prostitution (buying or selling) charges were clients of prostitute women. (Manhattan's very low percentage, 51%, may stem from its much higher volume of prostitution arrests per year than other jurisdictions and likely higher proportion of male/transvestite prostitutes). We defined clients in the Virginia data as men charged with patronizing specifically or, when the arrest offense was listed as a nonspecific prostitution charge, according to the rule we used for Texas. The Connecticut records included only patronizing convictions.

We sought prostitution arrest records from all cities in Washington state with populations greater than 25,000 residents (or counties with an incorporated city with more than 15,000 residents). Most jurisdictions and arrests indicated patronizing specific charges, but for those few that listed only nonspecific prostitution charges, we applied the rule for defining clients that we used for Texas. The ten jurisdictions that provided suitable data and the years covered by the data were the police departments of Bellingham (1997–2003), Bremerton (1996–2003), Federal Way (1997–2003), Lakewood (2002–4), Lynnwood (1996–2003), Renton (1998–2003), Seattle (1949–2004), Tacoma (2002–4), and Yakima (1981–2003) and the sheriff's offices of King (1998–2003) and Pierce (2002–4) counties. For the Yakima arrest data, we modified the Texas rule for defining clients by treating men arrested for prostitution on dates with 3 or more such male arrests as clients. Yakima has a small population (71,845 in the 2000 Census) and the possibility of many male prostitutes working there on the same day seems remote. (Indeed, in the other small Washington cities that have data on specific prostitution charges [Bremerton and Lakewood], all males arrested on dates when 3 or more males were arrested on prostitution charges were clients of prostitute women). The jurisdictions with known proactive vice operations against clients that did not respond to our requests or were unable or unwilling to provide suitable data were the police departments of Edmonds, Everett, Fife, Kent, Pasco, and Spokane, and the Spokane County Sheriff's Office.

To investigate displacement of patronizing from the street to off-street sectors of prostitution, we obtained patronizing arrest records for Frederick and Hagerstown, Maryland, by searching the online archives of the newspaper serving the area (Herald-Mail; http://www.herald-mail.com), which has routinely published reports of arrests made by local police. We also acquired from the City of Frederick the records of clients who patronized a Frederick escort agency between September 3, 1996, and December 2, 1999. This agency served as a main source of off-street prostitution in the area of these cities during this period. These records were made public as a result of criminal judicial proceedings against the agency's owner. The list of agency clients includes only first and last names and no further identifying information.

## Results

### Comparability of police- and public health-detected clients

Police- and public health-detected clients were similar in terms of demographics, locality of residence, and patronizing behavior. Police-detected clients from 1970–2000 were, on average, several years younger than public health-detected clients from 1985–2000 (Table 1). However, this difference may be due to a cohort effect (increasing average age of clients over time), because the difference nearly vanishes for police- and public health-detected clients drawn from the same 1985–2000 period. Similarly, the slight differences in race and active Army status between all police-detected and public health-detected clients disappear when the comparisons are restricted to those identified in 1985–2000 (Tables 2 and 3). Table 4 shows that public health-detected clients were mildly more likely than police-detected clients to reside locally (i.e., within El Paso and Teller Counties, the service area for the local health department). Other analyses, detailed in the following section, though, suggest that clients who were local residents were not more likely to be rearrested than clients who resided elsewhere.

**Table 1:**
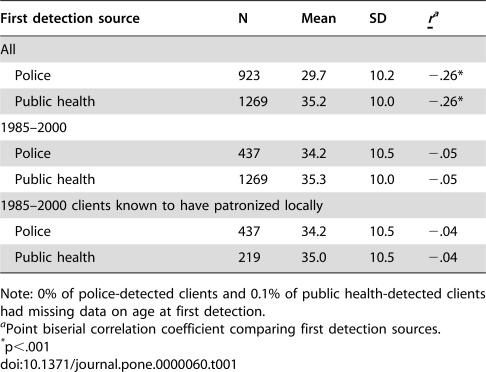
Clients' Age at First Detection by First Detection Source

First detection source	N	Mean	SD	*r* a
All				
Police	923	29.7	10.2	−.26*
Public health	1269	35.2	10.0	−.26*
1985–2000				
Police	437	34.2	10.5	−.05
Public health	1269	35.3	10.0	−.05
1985–2000 clients known to have patronized locally				
Police	437	34.2	10.5	−.04
Public health	219	35.0	10.5	−.04

Note: 0% of police-detected clients and 0.1% of public health-detected clients had missing data on age at first detection.

a Point biserial correlation coefficient comparing first detection sources.

* p<.001

**Table 2:**
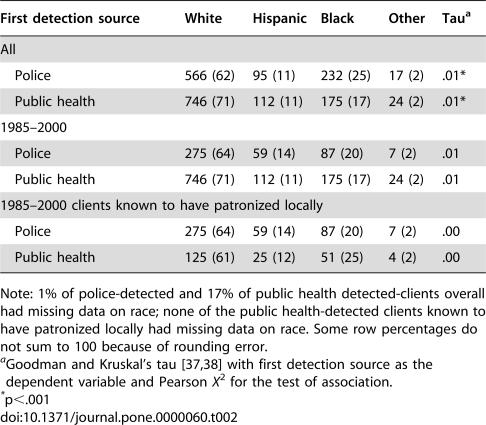
Crosstabulation of First Detection Source by Race (Row Percentages in Parentheses)

First detection source	White	Hispanic	Black	Other	Tau^a^
All					
Police	566 (62)	95 (11)	232 (25)	17 (2)	.01*
Public health	746 (71)	112 (11)	175 (17)	24 (2)	.01*
1985–2000					
Police	275 (64)	59 (14)	87 (20)	7 (2)	.01
Public health	746 (71)	112 (11)	175 (17)	24 (2)	.01
1985–2000 clients known to have patronized locally					
Police	275 (64)	59 (14)	87 (20)	7 (2)	.00
Public health	125 (61)	25 (12)	51 (25)	4 (2)	.00

Note: 1% of police-detected and 17% of public health detected-clients overall had missing data on race; none of the public health-detected clients known to have patronized locally had missing data on race. Some row percentages do not sum to 100 because of rounding error.

a Goodman and Kruskal's tau [37], [38] with first detection source as the dependent variable and Pearson *X* ^2^ for the test of association.

* p<.001

**Table 3:**
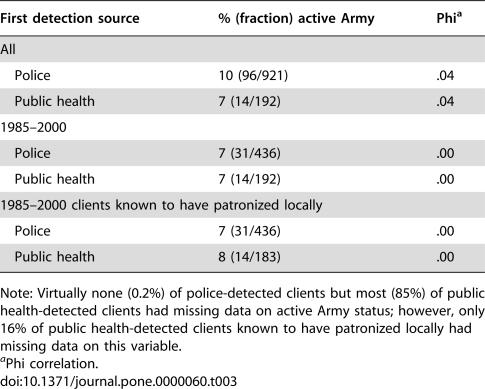
Summary of First Detection Source by Active Army Status

First detection source	% (fraction) active Army	Phi^a^
All		
Police	10 (96/921)	.04
Public health	7 (14/192)	.04
1985–2000		
Police	7 (31/436)	.00
Public health	7 (14/192)	.00
1985–2000 clients known to have patronized locally		
Police	7 (31/436)	.00
Public health	8 (14/183)	.00

Note: Virtually none (0.2%) of police-detected clients but most (85%) of public health-detected clients had missing data on active Army status; however, only 16% of public health-detected clients known to have patronized locally had missing data on this variable.

a Phi correlation.

**Table 4:**
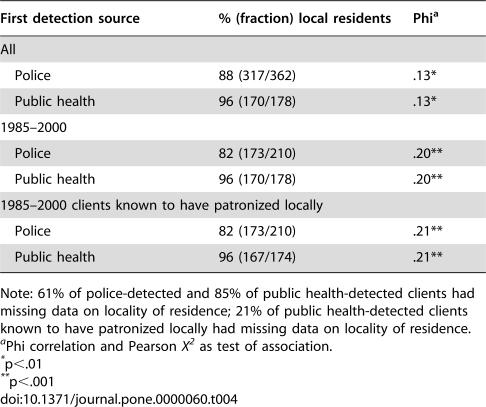
Summary of First Detection Source by Locality of Residence

First detection source	% (fraction) local residents	Phi^a^
All		
Police	88 (317/362)	.13*
Public health	96 (170/178)	.13*
1985–2000		
Police	82 (173/210)	.20**
Public health	96 (170/178)	.20**
1985–2000 clients known to have patronized locally		
Police	82 (173/210)	.21**
Public health	96 (167/174)	.21**

Note: 61% of police-detected and 85% of public health-detected clients had missing data on locality of residence; 21% of public health-detected clients known to have patronized locally had missing data on locality of residence.

a Phi correlation and Pearson *X^2^* as test of association.

* p<.01

** p<.001

The available evidence also suggests that police- and public health-detected clients were comparable in terms of patronizing behavior. Four clients of prostitute women who were interviewed in the study of local prostitutes, drug injectors, and their partners were first arrested for patronizing in Colorado Springs after participating in the study. Three of these clients reported the number of prostitutes in Colorado Springs they had patronized in the 5 years before the study. The mean and median numbers reported by these clients (4.3 and 3.0) were close to those reported by clients of prostitute women in the study who were never arrested for patronizing in Colorado Springs during the observation period (n = 114, mean = 7.3, median = 3.0).

### Comparing rates of (re)arrest

**Figure 1:**
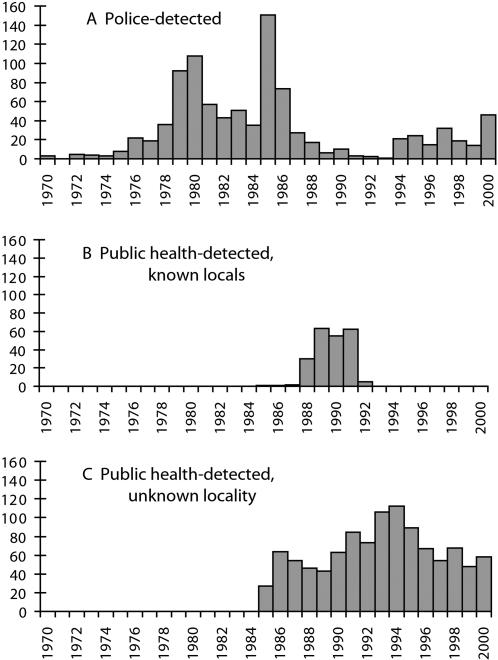
(A) Police-detected Clients. (B) Public Health-detected Clients Known to Have Patronized Locally. (C) Public Health-detected Clients Whose Patronizing Locality was Unknown.

Figure 1 displays the distribution of first detections over time by source. According to Colorado Springs police, the dip in number of arrests in 1992–3 was due to diverting police effort toward enforcement against the crack cocaine trade, and the decline in patronizing arrests in the late 1990s was a result of increasingly charging clients with indecent exposure (catching them exposed in public while patronizing) rather than conducting stings and charging clients with prostitution.

Because police ascertainment of clients began before public health identification of clients, we sought to eliminate different lengths of observation from confounding our analyses. For the period of overlapping police and public health surveillance of clients (1985–2000), police-detected and public health-detected clients known to have patronized locally both had 10.5 person-years of observation on average. Therefore, we constructed a moving cohort of clients first detected by the police before 1985, and followed each for a 10.5 year observation window subsequent to his arrest. Our calculations of incidence are based on following a client until he was arrested for the first time (for public health-detected clients) or rearrested (for police-detected clients). Each client who was not arrested or rearrested was followed until either the end of his 10.5 year observation window or the end of 2000 (when all active observations were censored).

The rearrest rate for police-detected clients is just a fraction of the arrest rate for public health-detected clients, although both rates are quite low in absolute terms (Tables 5 and 6). The ratios of the crude rates range from 0.29 to 0.48 for different sets/subsets of police- and public health-detected clients. The risk of arrest, however, changed over the observation period, as illustrated by fluctuations in the number of clients arrested (Figure 1). Therefore, we measured the time-varying risk of arrest that a given set of clients faced by the mean number of arrests in the person-years observed for those clients. When the arrest rates are adjusted to account for the risk of arrest (increasing the public health-detected clients' rate proportionate to the police-detected clients' higher risk of arrest), the rate ratios decrease to 0.14–0.43.

**Table 5:**
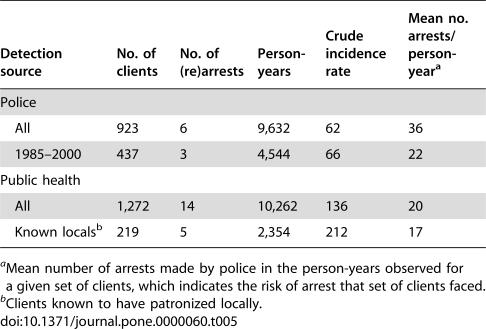
Crude Incidence Rates per 100,000 Person-Years of (Re)Arrest for Police- and Public Health-Detected Clients

Detection source	No. of clients	No. of (re)arrests	Person-years	Crude incidence rate	Mean no. arrests/person-year^a^
Police					
All	923	6	9,632	62	36
1985–2000	437	3	4,544	66	22
Public health					
All	1,272	14	10,262	136	20
Known locals^b^	219	5	2,354	212	17

a Mean number of arrests made by police in the person-years observed for a given set of clients, which indicates the risk of arrest that set of clients faced.

b Clients known to have patronized locally.

**Table 6:**
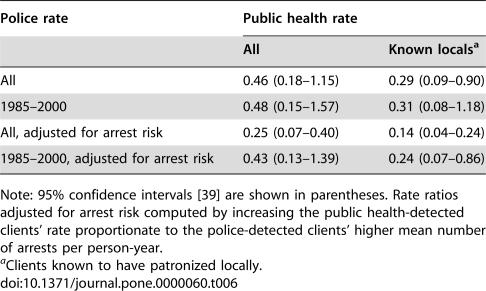
Ratios of Police-Detected Clients' Rearrest Rate to Public Health-Detected Clients' Arrest Rate

Police rate	Public health rate	
	All	Known locals^a^
All	0.46 (0.18–1.15)	0.29 (0.09–0.90)
1985–2000	0.48 (0.15–1.57)	0.31 (0.08–1.18)
All, adjusted for arrest risk	0.25 (0.07–0.40)	0.14 (0.04–0.24)
1985–2000, adjusted for arrest risk	0.43 (0.13–1.39)	0.24 (0.07–0.86)

Note: 95% confidence intervals [39] are shown in parentheses. Rate ratios adjusted for arrest risk computed by increasing the public health-detected clients' rate proportionate to the police-detected clients' higher mean number of arrests per person-year.

a Clients known to have patronized locally.

The difference in arrest rates does not appear to be due to the modest difference between police- and public health-detected clients in local residence. Fifty-three clients (45 police-detected, 8 public health-detected) were known to have resided outside the local area. One of the 45 police-detected clients was rearrested, and none of the eight public health-detected clients was arrested. One of 317 police-detected clients who were known to reside locally was rearrested. The crude rearrest rate is much lower for local residents (26 per 100,000 person-years) than for nonlocal residents (160 per 100,000 person-years) despite more arrests in the observed person-years for the local residents (mean = 39) than nonlocal residents (mean = 31). The rearrest rates, adjusted for arrest risk relative to police-detected clients overall, are 24 per 100,000 person-years for local residents and 186 per 100,000 person-years for nonlocal residents. Although these estimates may be unreliable because they are each based on a numerator of one, it seems unlikely that locality of residence accounts for much of the large difference in (re)arrest rates by first detection source.

The similarity of the crude rearrest rates for all police-detected clients and those first detected between 1985 and 2000 (Table 5) is somewhat unexpected given that these sets of clients faced substantially different risks of rearrest on average. However, the corresponding adjusted rates are within the range of sampling variability. The estimated incidence rate of rearrest for clients first detected by the police between 1985 and 2000, adjusted for arrest risk relative to all police-detected clients, is 108 per 100,000 person-years. The 95% confidence interval for this rate [24], [25], 46 to 255 per 100,000 person-years, includes the estimated incidence rate of rearrest for all police-detected clients, 62 per 100,000 person-years. An increase in the rearrest rate could signal a decrease in client prevalence over time. However, it is unlikely that the population of local clients was larger before 1985 because the prevalence of prostitute women in Colorado Springs showed no discernible declining trend in the 1980s [26]. Even if the proportion of Colorado Springs men who were clients declined during the observation period, the absolute number of local clients likely would not have decreased, as the overall county population increased from 235,972 in 1970 to 516,929 in 2000 (http://www.factfinder.census.gov).

### Survival analysis of the specific deterrent effect of arrest

We also estimated the specific deterrent effect of arrest with discrete-time survival analysis models [27], [28] (Table 7). Each model includes first detection source, discrete time and time^2^ (representing the possibility of an inverted U-shaped risk of arrest over time due to outmigration, behavior change, death, etc.), and the natural logarithm of the number of arrests in a person-year. The natural logarithm of arrests term represents a potential multiplicative relationship with (re)arrest in the same way our adjustments of the rate ratio for arrest risk do. We added one arrest for the year 1971 to allow calculation of the natural logarithm of the number of arrests for each year in the observation period. The survival analysis results should be treated as approximate, because this analytic approach is sensitive to small numbers of events (few (re)arrests in our case).

**Table 7:**
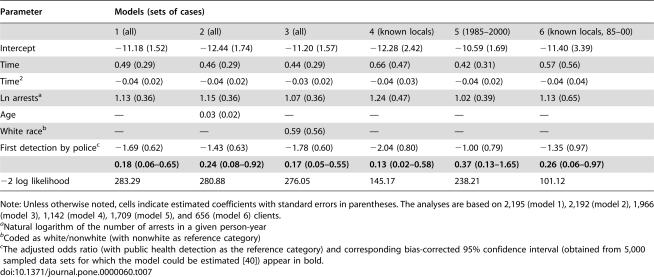
Survival Analysis Results

Parameter	Models (sets of cases)					
	1 (all)	2 (all)	3 (all)	4 (known locals)	5 (1985–2000)	6 (known locals, 85–00)
Intercept	−11.18 (1.52)	−12.44 (1.74)	−11.20 (1.57)	−12.28 (2.42)	−10.59 (1.69)	−11.40 (3.39)
Time	0.49 (0.29)	0.46 (0.29)	0.44 (0.29)	0.66 (0.47)	0.42 (0.31)	0.57 (0.56)
Time^2^	−0.04 (0.02)	−0.04 (0.02)	−0.03 (0.02)	−0.04 (0.03)	−0.04 (0.02)	−0.04 (0.04)
Ln arrests^a^	1.13 (0.36)	1.15 (0.36)	1.07 (0.36)	1.24 (0.47)	1.02 (0.39)	1.13 (0.65)
Age	—	0.03 (0.02)	—	—	—	—
White race^b^	—	—	0.59 (0.56)	—	—	—
First detection by police^c^	−1.69 (0.62)	−1.43 (0.63)	−1.78 (0.60)	−2.04 (0.80)	−1.00 (0.79)	−1.35 (0.97)
	**0.18 (0.06–0.65)**	**0.24 (0.08–0.92)**	**0.17 (0.05–0.55)**	**0.13 (0.02–0.58)**	**0.37 (0.13–1.65)**	**0.26 (0.06–0.97)**
−2 log likelihood	283.29	280.88	276.05	145.17	238.21	101.12

Note: Unless otherwise noted, cells indicate estimated coefficients with standard errors in parentheses. The analyses are based on 2,195 (model 1), 2,192 (model 2), 1,966 (model 3), 1,142 (model 4), 1,709 (model 5), and 656 (model 6) clients.

a Natural logarithm of the number of arrests in a given person-year

b Coded as white/nonwhite (with nonwhite as reference category)

c The adjusted odds ratio (with public health detection as the reference category) and corresponding bias-corrected 95% confidence interval (obtained from 5,000 sampled data sets for which the model could be estimated [40]) appear in bold.

In the base model, the adjusted odds ratio for first detection source (with public health as the reference category) for all clients is 0.18 (95% CI 0.06–0.65), indicating a strong specific deterrent effect of arrest. Models analogous to the other comparisons in Table 6 and those that also included race or age have adjusted odds ratios for first detection source ranging from 0.13 to 0.37 (Table 7). In these latter models, the associations between age and race and (re)arrest are slight. The substantial independent relationship between the natural logarithm of number of arrests in a person-year and (re)arrest in all models underlines the necessity of adjusting the rate ratios for arrest risk. We did not estimate models that included active Army status or locality of residence because the loss of sample size (including (re)arrest events) from missing data was too severe.

### Assessing displacement

Data from other parts of the US suggest that our main result is not likely caused by displacement of arrested clients' patronizing to other jurisdictions or sectors of prostitution. Table 8 shows that only a very few clients rearrested for patronizing in 4 states were arrested in multiple local jurisdictions, and many of these resided in the arrest jurisdiction at each arrest (i.e., they moved their residence from one arrest jurisdiction to another). Thus, the share of rearrested clients whose patronizing could possibly have been displaced geographically seems to be less than 10%. Some clients so classified may not have actually been displaced, as the multiple arrest jurisdictions could reflect their pre-existing ranges for patronizing.

**Table 8:**
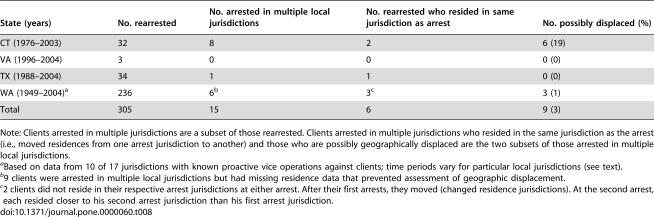
Summary of Rearrested Clients whose Patronizing Possibly Could Have Been Displaced from One Jurisdiction to Another Subsequent to Arrest

State (years)	No. rearrested	No. arrested in multiple local jurisdictions	No. rearrested who resided in same jurisdiction as arrest	No. possibly displaced (%)
CT (1976–2003)	32	8	2	6 (19)
VA (1996–2004)	3	0	0	0 (0)
TX (1988–2004)	34	1	1	0 (0)
WA (1949–2004)^a^	236	6^b^	3^c^	3 (1)
Total	305	15	6	9 (3)

Note: Clients arrested in multiple jurisdictions are a subset of those rearrested. Clients arrested in multiple jurisdictions who resided in the same jurisdiction as the arrest (i.e., moved residences from one arrest jurisdiction to another) and those who are possibly geographically displaced are the two subsets of those arrested in multiple local jurisdictions.

a Based on data from 10 of 17 jurisdictions with known proactive vice operations against clients; time periods vary for particular local jurisdictions (see text).

b 9 clients were arrested in multiple local jurisdictions but had missing residence data that prevented assessment of geographic displacement.

c 2 clients did not reside in their respective arrest jurisdictions at either arrest. After their first arrests, they moved (changed residence jurisdictions). At the second arrest, each resided closer to his second arrest jurisdiction than his first arrest jurisdiction.

The available evidence also suggests that displacement of arrested clients' patronizing to off-street prostitution may have been rare. Eighty-nine men were arrested for patronizing on the street in the cities of Frederick and Hagerstown, Maryland, during the period of the Frederick escort agency records. Nine hundred thirty-one clients appear in the agency records. Only one client arrested in this period was listed in the escort agency records and his first encounter with the agency predated his arrest for patronizing on the street. There were 152 person-years in total between the arrested clients' arrest dates and the end of the period of the escort agency's records.

## Discussion

We compared clients of prostitute women in Colorado Springs first detected by the police and those first detected by public health in terms of their rates of arrest. Our analyses indicate that arrest reduces the likelihood of a future patronizing arrest by about 70%. Clients first detected by the two sources were similar in demographics, locality of residence, and patronizing behavior, and these factors could not account for the large difference in arrest incidence by first detection source. Moreover, evidence from other parts of the US indicates little displacement of patronizing to other jurisdictions or sectors of prostitution following an arrest for patronizing a street prostitute. Taken together, our results suggest that apprehending clients decreases their patronizing behavior substantially.

Our findings contrast starkly with prior reports of no specific deterrent effect of arrest among young offenders for other types of offenses [11]–[15]. Arrest may be a significant deterrent for clients because they generally are otherwise law-abiding men [29] (Brewer et al., unpublished data) who could suffer loss of reputation and marital or romantic relationship conflict as a consequence of arrest. Such themes are often apparent in clients' comments at arrest, both as others have noted [30], [31] and we have observed in arrest narratives from several jurisdictions. Our results also suggest that arrest, with the attendant criminal and judicial processing, typically did not cause clients to internalize an official label of “client” that served to perpetuate their patronizing [32]. Labeling might not have occurred because one key element thought to be crucial in the labeling process—association with deviant groups following official processing [32]—may be absent, as clients seem to interact rarely with each other as clients. In fact, 75% of arrested clients in an Edmonton sample had never told anyone about their patronizing behavior [33].

Given the large specific deterrent effect of arrest for patronizing, any special post-arrest intervention or extra penalty for patronizing may not have a noticeable impact, as there may be little additional deterrence that could be achieved. Indeed, convicted clients who attended “john school” (a program where clients are presented with information on the harms of prostitution to prostitutes, communities, and clients) following a court order in Portland, Oregon, had a similar patronizing reconviction rate as temporally-matched convicted clients who were not ordered to attend but were apparently otherwise similar [29]. Similarly, john school in Toronto did not change clients' intentions to patronize in the future, which were already quite low after arrest but before john school [34].

The low rate of recidivism we observed in both groups was produced mostly by the low absolute risk of arrest and primarily reflects the large population of clients [35]. Specific deterrence probably has a limited impact on the overall prevalence of clients as we estimate that only 7–18% of clients in a community are ever arrested for patronizing over periods as long as 5 years (Brewer et al., unpublished data). Colorado Springs detectives independently reported to us their perception that arrest had a substantial specific deterrent effect but only a mild, temporary effect on overall local patronizing activity. Nonetheless, active and potential clients' awareness of the law against patronizing and the possibility of its enforcement likely promote general deterrence, even though most vice operations are conducted covertly and not well-publicized (in Colorado Springs and many US communities). Indeed, the introduction of a law against patronizing in Sweden and enforcement of it appears to have dramatically reduced street prostitution, based on informal assessments [36]. Priority topics for future investigation include general replication of our findings, evaluation of whether expanded, intensified, and high profile enforcement of laws against patronizing can reduce the level of prostitution further, and examination of the specific deterrent effect of arrest for other offenses.
